# Application of Gelatin in Food Packaging: A Review

**DOI:** 10.3390/polym14030436

**Published:** 2022-01-21

**Authors:** Yanan Lu, Qijun Luo, Yuchan Chu, Ningping Tao, Shanggui Deng, Li Wang, Li Li

**Affiliations:** 1 Engineering Research Center of Food Thermal-Processing Technology, College of Food Science and Technology, Shanghai Ocean University, Shanghai 201306, China; lyn13140176892@163.com (Y.L.); L1620438004@163.com (Q.L.); wh_nj_hf@163.com (Y.C.); nptao@shou.edu.cn (N.T.); 2 Engineering Research Center of Food Thermal Processing Technology, College of Food and Pharmacy, Zhejiang Ocean University, Zhoushan 316000, China; dengshanggui@163.com

**Keywords:** gelatin packaging, edible film and coating, modification, fresh application

## Abstract

Owing to the increasing environmental concerns and requirements for high-quality foods, edible films and coatings (based on proteins, polysaccharides, natural phenolic active substances, etc.) are being developed as effective alternatives to traditional plastic packaging. Gelatin is extracted from collagen. It is an ideal material for food packaging due to its versatile advantages such as low price, polymerization, biodegradability, good antibacterial and antioxidant properties, etc. However, gelatin film exists poor waterproof and mechanical properties, which limit its developments and applications in food packaging. Previous studies show that pure gelatin can be modified by adding active ingredients and incorporating them with bio-polymers to improve its mechanical properties, aiming to achieve the desirable effect of preservation. This review mainly shows the preparation and molding ways of gelatin-based edible films and the applications of gelatin modified with other biopolymers. Furthermore, this review provides the latest advances in gelatin-based biodegradable packaging and food applications that exhibit outstanding advantages in food preservation.

## 1. Introduction

The concept of edible films/coatings comes from the environmental pollution issues of dealing with non-biodegradable plastics and the demands of consumers for natural, nutritious and healthy food. Edible films/coatings are thin layers of edible materials, which can alter not only the molecular exchange between food and the environment but also different compartments of the same food to aid in food freshness, transportation, storage and display when applied to food [1]. Meat, fish and derivatives are perishable foods that can deteriorate rapidly under specific storage conditions (e.g., high storage temperature and relative humidity). Edible films and coatings with enhanced functions have effectively controlled the quality deterioration of fish, meat and derivatives (mainly by inhibiting microbial growth, antioxidant activity and maintaining sensory properties) [2]. Besides, edible biocomposite films and coatings have a great potential for shelf life extension and quality control for tropical fruits, berries and other fruits and vegetables by reducing weight loss, preventing color changes, respiration rate, controlling the ethylene and delaying the ripening of fruits and vegetables [3]. With the demand for healthy food, numerous new materials are being widely explored as raw materials for edible films and coatings.

**Figure 1:**
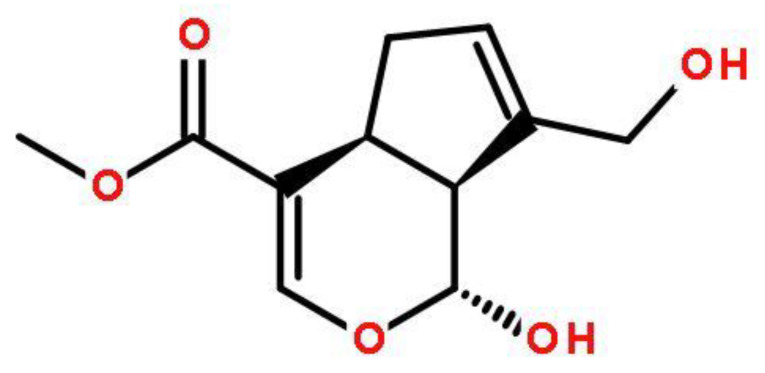
Chemical structure of gelatin.

Gelatin, a white or yellowish, translucent, lustrous solid, is a partially degraded product of collagen in animal connective tissue (Figure 1). Gelatin can be widely used in the food, pharmaceutical, photography and cosmetics industries due to its functional characteristics, including water-binding ability, gel formation ability, water vapor barrier, film forming, foam forming ability and emulsification tendency [4]. The gelatin shows strong gas barrier properties and swelling behavior in water; however, it suffers from weak mechanical resistance and permeability to water vapor molecules [5]. So, the use of gelatin as a packaging material is limited because of its weak water vapor barrier property [6]. This can be improved by mixing gelatin with other functional materials and active ingredients [7]. Previous studies focused on using proteins, carbohydrates, and phenols to improve the physical and biological properties of gelatin.

Edible films and coatings can be prepared by several techniques, including solution casting, extrusion, coating, layer-by-layer assembly (LBL), and so forth. Solution casting, one of the most widely used methods for preparing edible films and coatings, is a relatively economical and simple method, during which intermolecular electrostatic and hydrogen bond formation results in the polymer structure [8]. Although extrusion, a promising approach for the fabrication of the films, is often preferred over solution casting methods due to faster processing time and lower energy consumption, there are a limited number of studies related to the use of this technology on gelatin film [9,10]. Coating is often applied on the surface of fresh foods such as fruits and vegetables, fish, meat, and so forth, for enhancing their shelf-life [11]. LBL self-assembly can be used to prepare edible active packaging films and coatings by the incorporation of active agents either between layers or within the structure of an individual polyelectrolyte [12]. For example, 3-polylysine and polyglutamic acid are deposited onto water-soluble sugar–cargo mixtures by an organic solvent-based assembly technique and are crosslinked. Cargo-loaded edible microcapsules are created by adding water to dissolve the sacrificial sugar template [13].

The present article provides a comprehensive review of types, preparation methods, molding methods of gelatin, the possibilities as an edible film/coating material and the applications in the preservation of various foods. The current study highlights the appropriateness of gelatin as a film/coating material, in combination with other biopolymers. The challenges, limitations, and prospects of the applications of gelatin for future studies are also discussed.

## 2. Types of Gelatin

Gelatin can be extracted from animal sources or organs by using different extraction methods/parameters (e.g., acid treatment, base treatment, enzyme treatment, extraction temperature, extraction time). Animal-derived organs and extraction methods/parameters have a great influence on the molecular weight and amino acid composition of the gelatin obtained, which determines the molecular structures (second, third and fourth structures), physicochemical properties and functional properties of gelatin [14].

**Figure 2:**
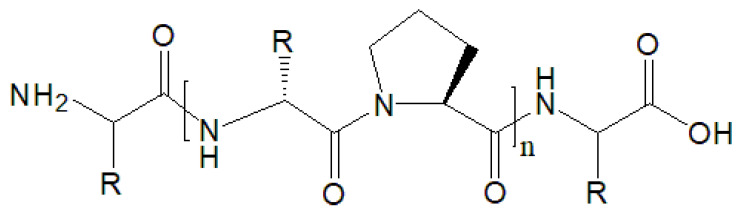
Chemical structure of collagen.

According to the sources of gelatin obtained by controlling hydrolysis of collagen, gelatin is divided into three main types of collagen: type I collagen, type II collagen and type III collagen. Type I collagen is found primarily in connective tissues, such as skin, bone and tendon, unique in forming a right-handed triple super helical structure consisting of three alpha (similar size) left-handed helical polypeptide chains. The collagen chain is characterized by Glycine-X-Y repeating motifs, in which X and Y positions are mainly occupied by Pro and Hyp. The Type II, Type III and other types of gelatin are found in cartilage tissue, very young skin and organs, respectively [4]. The chemical structure of collagen is shown in Figure 2.

Gelatin can be divided into two types according to the production process: type A gelatin and type B gelatin. The gelatin products obtained by acid extraction are called A gelatin, with an isoelectric point in 6~9, being the most used among the covalently bound collagen with less cross-linking degree recognized by pig skin. Alkali-extracted gelatin products are type B, with an isoelectric point of pH 5, which can be applied to the more complex collagen found in cow hide [4,15]. Compared to type A gelatin, nanoparticles in type B gelatin have a higher degree of cross-linking and therefore degrade more slowly. Besides, type B gelatin nanoparticles have a variety of physical and chemical properties, including film-forming properties, emulsification, and foaming properties, and so forth. These properties influence the amide groups of asparagine and glutamate, utilizing hydrolysis of the carboxyl groups, releasing and interacting to convert many of these residues into various compounds of asparagic acid and glutamate. In general, gelatin has both acidic and alkaline functions due to its functional amino acid group, terminal amino acid group and carboxyl group [16].

**Figure 3:**
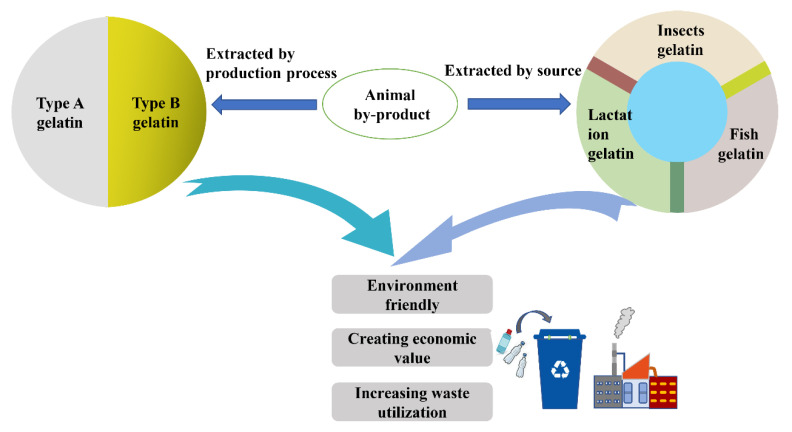
Schematic representation to extract gelatin according to different methods and sources and realize circulation for gelatin films.

According to different sources, gelatin can be divided into lactation gelatin species (e.g., cattle and pigs), fish gelatin of aquatic species (e.g., cold-water fish and warm-water fish) and insect gelatin species (e.g., sorghum adult and melon adult) [17]. Gelatin can be extracted by the hydrolysis of collagens from animal sources, but not plants. Some so-called “vegetable gelatins”, such as seaweed gelatins, are not classified as gelatins because they have no chemical relationship with gelatin [16]. Traditionally, gelatin is obtained from cow hide, pig hide, demineralized bones and hooves [14]. However, recently for religious reasons, people, such as Jews and Muslims, do not accept foods made from mammalian gelatin. In addition, the emergence of human and animal infections (e.g., bovine spongiform encephalopathy, transmissible spongiform encephalopathy, foot-and-mouth disease, etc.) has also aroused people’s concerns about mammals that may be infected with these diseases [18]. Therefore, the gelatin community and industry show great interest in developing alternative gelatins from non-mammals such as fish and insects [19]. The fish processing industry in the fillet production process, with a product yield of only 30–50%, can extract gelatin from a large number of by-products from the bones and skin [20]. The classification of different extraction methods is shown in Figure 3. Fish gelatin is considered a promising alternative to mammals’ gelatin that can create economic value for fish by-products (account for 70% of the total weight of fish [21]) and reduce the seafood industry’s waste.

## 3. The Preparation Methods of Gelatin-Based Edible Composite Films and Coatings

**Figure 4:**
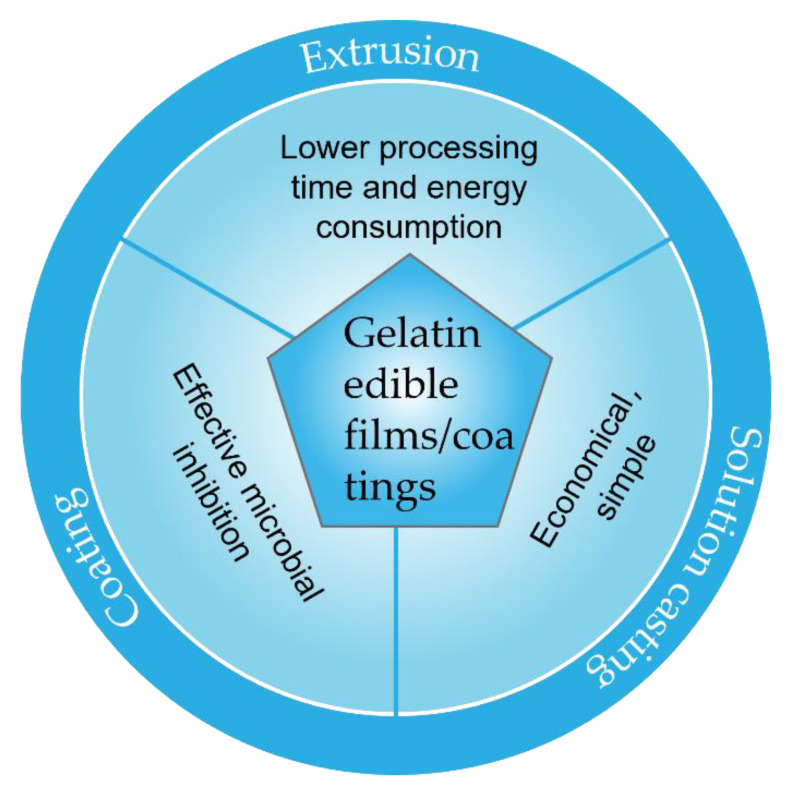
Common techniques for producing edible films and coatings.

Fabrication techniques for gelatin-based edible composite films and coatings are shown in Figure 4.

### 3.1. Solution Casting

The solution casting method is a molding method that is used to dissolve biopolymers and is mixed with plasticizers or additives to produce composite films by preparing a film solution, which is widely used in the food packaging industry to develop a gelatin-based composite films [22]. Chitosan is a soluble form of chitin and has been used in various industrial applications including uses in food preservation and packaging. Studies showed that chitosan gelatin-based composite films are prepared by the solution casting method [23]. Roy et al. reported steps of solution casting of gelatin with chitosan. Chitosan and gelatin were added to an aqueous solution of 1% acetic acid in equal proportions, stirring and heating to dissolve before cooling to room temperature. Tween 80 and 2 wt % cinnamon essential oil (CEO) were dispersed in distilled water and stirred vigorously, adding 1.0 wt % rutin (biopolymer-based) to the membrane forming solution and stir. The film forming solution was casted on a flat Teflon film-coated glass plate (24 cm × 30 cm) and dried at room temperature for 48 h before peeling. Chitosan/gelatin-based functional films with strong antibacterial and antioxidant activity are available for active packaging application [24]. The developed film showed an improved ultraviolet ray (UV) blocking properties, mechanical properties, antioxidant and antibacterial activities. Musso et al. prepared a gelatin–curcumin composite film based on (0.4 wt %) gelatin containing only (0.02 vol %) curcumin by the solution casting method and tested the pH response and oxidation resistance of the composite film. It was found that the film showed high antioxidant activity and susceptibility to media pH. The developed film can be used as smart food packaging as it could provide information about food spoilage indirectly through media pH measurement and extend the shelf-life of food through the material’s antioxidant properties [25].

### 3.2. Extrusion

**Figure 5:**
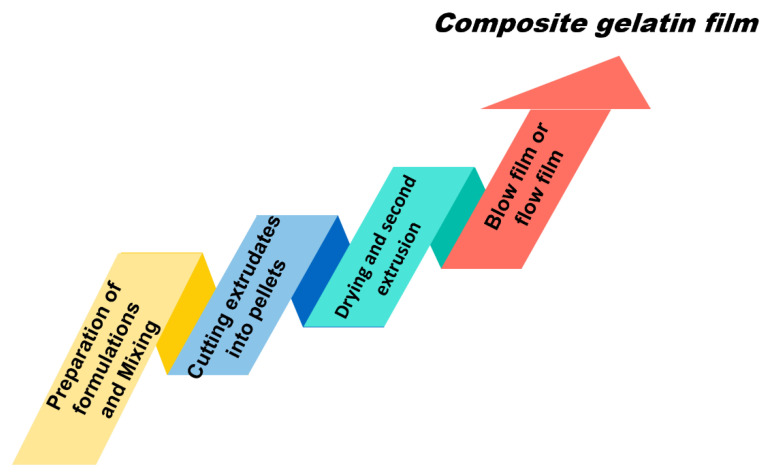
Steps for the preparation of edible composite film for the extrusion method.

In plastic processing, extrusion molding is a processing method of being made into a variety of cross section products or semi-products, which refers to the material being through the role between the extruder barrel and the screw, one side heating plasticizing, one side being pushed forward by the screw. The extrusion method is mainly used to produce conventional commercial plastic packaging films. Generally, because of its high production efficiency and low energy consumption, it is widely used in the processing of rubber, plastic and fiber, so as to be more popular than solution casting [9]. Cheng et al. mixed gelatin with the lake for 5 min at room temperature and homogenized glycerol, deionized water, beeswax and Tween 80 with the prepared starch/gelatin mixture for another 10 min. The screw speed was set to 125 rpm and the extrusion temperature from barrel to die was set to 90, 100, 105, 110, 100 and 90 °C. Before blowing the film, the extrudates were cut into particles, then regulated at 53 ± 2% relative humidity and 23 ± 2 °C for at least 72 h [26]. Although the processing time by extrusion molding is faster and the efficiency is higher, compared to other forming ways, the studies on gelatin extrusion are relatively limited. More studies are encouraged to explore the possibility and efficacy of the production of gelatin film by the extrusion method. A schematic representation of particular steps for the preparation of gelatin-based edible composite film for the extrusion method is shown in Figure 5.

### 3.3. Coating

Edible coatings are usually applied in liquid form by dipping or spraying fruits and vegetables to form a coating on the surface [27]. Edible coatings are usually composed of film-forming ingredients and additives. A variety of naturally derived polymers are effectively used as film forming components in the formulation of food coatings such as proteins, polysaccharides and lipids. Gelatin, as a biopolymer extracted from collagen, has been widely used as a film-forming component of edible coatings and used to keep fresh persimmon, tomato, cherry, bread and other fresh foods [28,29]. Carboxymethyl chitosan (CMCS) solution and gelatin at 2 % (*w*/*v*) solution were prepared separately in distilled water in a 60 °C water bath. The mixture was continuously stirred for 30 min until it was completely dissolved before cooling to 23 ± 1 °C. CMCS was mixed with gelatin in a ratio of 2:1 at 23 ± 1 °C. The mixture was stirred for 30 min in the presence of 1% glycerol and 0.1% Tween 20 acting as plasticizer and surfactant, respectively. Finally, the mixture was centrifuged to remove air bubbles and particulates, and the supernatant was collected for the film preparation [29]. Chin et al. added 30% *wt*/*wt* of glycerol into the (6% *wt*/*v*) gelatin dispersion. Aloe vera extraction was added into the gelatin dispersion at different concentrations (1–9% *wt*/*wt*) to prepare coating films. The composite coating increased significantly in solubility and plasticity. Besides, the coating showed enhanced mechanical properties with the increase of Aloe gel concentration [30]. Another study has shown that furcellanan and gelatin were mixed with water (0.5:1:98.5 *v*/*v*/*v*) for 30 min at 50 °C, followed by the addition of tea extract or water (in the case of control samples) with high antibacterial and antioxidant properties and glycerin (1% *v*/*w*). The mixture was stirred at 300 rpm for 15 min to obtain an edible coating solution [31].

## 4. Modified with Other Polymers and Active Ingredients

Gelatin, a protein found in animal skin and bones, is a mixture of peptides and proteins that shows gel-forming properties. Due to its good film-forming ability, emulsifying property and gas barrier property, it is commonly used in biodegradable films [32,33]. Gelatin can form a network of physical connections in a hybrid system. When plasticized properly, it can produce strong and flexible films [34]. However, native gelatin films become fragile if not in suitable temperature and humidity conditions (temperature at −10~40 °C, humidity in 50~70%), thereby becoming rigid, brittle, and exhibiting poor elongation at break (elongation at break <25%) [22]. In addition, the water resistance of gelatin film is very poor. When contacted with water, they may dissolve, expand or disintegrate. Therefore, mixing gelatin with other natural polymers and active ingredients is currently the most effective way to improve the functional properties of gelatin. Previous literature has reported that gelatin had been combined with chitosan, starch, soy protein isolate and carboxymethyl cellulose to improve the performance of packaging applications [35,36,37,38]. The main modified materials and physical and biological properties improved are shown in Table 1.

### 4.1. Gelatin Blended with Carbohydrate

**Figure 6:**
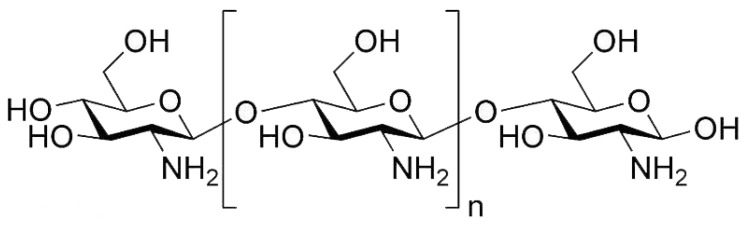
Chemical structure of chitosan.

Polysaccharides can be obtained from a wide range of sources, such as plant cell walls (e.g., cellulose, pectin and peptidoglycan), animals (e.g., chitin, hyaluronic acid and chondroitin) and microorganisms (e.g., xanthan gum and glucan) [49]. The presence of polysaccharides can modify the protein surface structure and weaken the formation of the protein network structure by increasing protein hydrolysis. Due to the Maillard reaction between protein and polysaccharide, the polymerization of gelatin and carbohydrate can not only improve the physical and chemical properties of gelatin film, but also change its biological properties significantly. For instance, chitosan (CHI) is a kind of polysaccharide, which is made of natural chitin deacetylated at high pH value and has good film-forming ability and antibacterial and antioxidant ability [50] (Figure 6). When CHI was mixed with gelatin, the content of β-folding decreased with the addition of CHI, while the content of α-helix and random curl increased and the solubility of the film decreased significantly. The contact angle value increased with the addition of CHI, resulting in the increase of surface hydrophobicity. The increased hydrophobicity of the film may be related to the interaction between polar groups shown by fourier transform infrared analysis, which obstructs the orientation of polar groups towards the surface. When CHI content increased, both tensile strength and elongation at break increased. In addition, chitosan–gelatin composite membrane had a good inhibitory effect on E coli [51]. Other studies have shown that the mixture of pigment (anthocyanin) and chitosan can result in more red and yellow colors of meat, reducing pH changes, inhibiting pork oxidation and microbial growth, and extending the shelf life of 20 days in cold storage [52].

### 4.2. Gelatin Modified with Enzymes and Proteins

Various enzymes and proteins can modify the chemical structure of gelatin, thereby affecting its mechanical properties, such as Glutaminase (MTGase, optimal activity at pH 5–8), tyrosinase and zein, etc. [22,53]. The study showed that, in the presence of soy protein isolate (SPI), the total soluble matter and protein solubility of gelatin film decreased from 89.36% and 92.78% to 35.83% and 40.05% after adding 3% transglutaminase (TGase), respectively, which may be due to the stronger solubility of gelatin in water than SPI. The strength of gelatin film was improved at 1% TGase, but decreased when the concentration of TGase was increased more. Moreover, the film has better thermal stability and water resistance [54]. The solubility and water vapor permeability of gelatin film greatly decreased at 0.1% (*w*/*v*) of the active casein phosphopeptides (CPPS). The lowest ultraviolet ray transmittance of the film was observed in the range of 200–350 nm, and the film showed improved mechanical properties and effective antioxidant activity. In general, gelatin-CPPS edible films with lower CPP concentrations (0.1–0.2% of CPP) exhibit the strongest physico-mechanical properties and bioactive characteristics [55]. Another study showed that TGase cross-linking and the addition of zein reduced WS in gelatin films and improved the mechanical strength and water insolubility required for most food packaging applications [53].

### 4.3. Gelatin Compounded with Polyphenols

**Figure 7:**
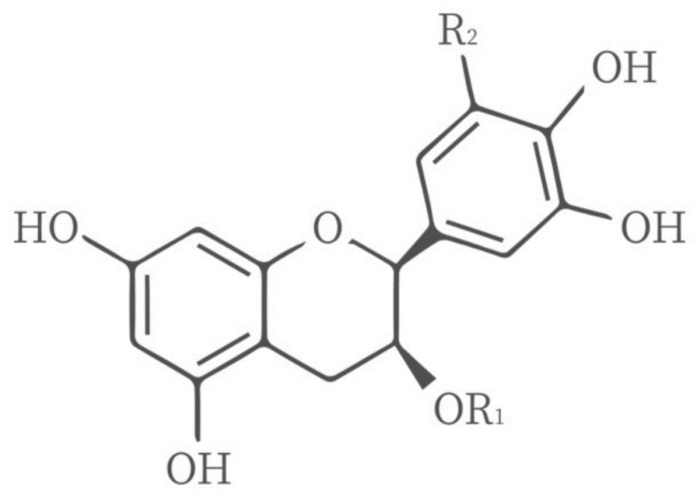
Chemical structure of tea polyphenols.

Polyphenols are a group of antioxidant chemicals extracted from plants (fruits, vegetables, grains, etc.) that can promote health, inhibit the activity of free radicals in the body and prevent damage to body tissues [54]. The chemical structure of polyphenols is shown in Figure 7. They are functionally and structurally diverse, with many types, such as tea polyphenols, phenolic acids, flavonoids, polyphenols amides, resveratrol and lignans [56]. A study added different concentrations of tea polyphenols (TP) to gelatin–sodium alginate (GSA) edible films and coatings to improve the physical morphology, antioxidant activity and structure of the gelatin film. The TP at low molecular weight is more easily inserted into the gelatin–sodium alginate network structure, resulting in covalent cross-linking and affecting the mechanical properties of GSA–TP films. Compared to GSA films, the water vapor permeability(WVP) of GSA films containing TP (0.8–2.0%) decreased significantly, which may be due to the dense system formed by the mosaic invasion of TP, which thus reduces the WVP. In addition, the light resistance and antioxidant activity of the film are significantly improved at 2.0% TP to prevent fat oxidation and extend the shelf life of food during storage [57]. In addition, the addition of other plant polyphenols, such as turmeric ethanol extract, grapefruit seed extract, chlorogenic acid, and so forth, can also improve the mechanical and antioxidant properties of gelatin films due to the cross-linking reaction between gelatin and polyphenols [58,59,60,61].

## 5. Application of Gelatin-Based Composite Edible Films and Coatings

**Figure 8:**
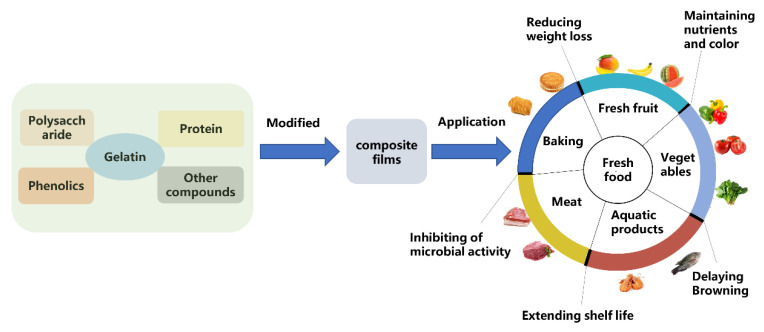
Modification and functional application of gelatin-based edible composite films in food preservation.

Nowadays, considering food safety’s impact on the health of consumers, the development of safe and hygienic edible coatings and films becomes more and more popular in the field of food packaging preservation. Biopolymers (e.g., proteins and polysaccharides) are used widely in the food industry to develop composite films and coatings due to their good biocompatibility, resulting in extended biological and technological functionality. Gelatin is compatible with several biopolymers that are commonly applied in edible coatings and films, which have been widely used in fresh products and improved the preservation value (Figure 8). The applications of gelatin-based films in various foods have been summarized and are shown in Table 2.

### 5.1. Fresh Fruit

**Figure 9:**
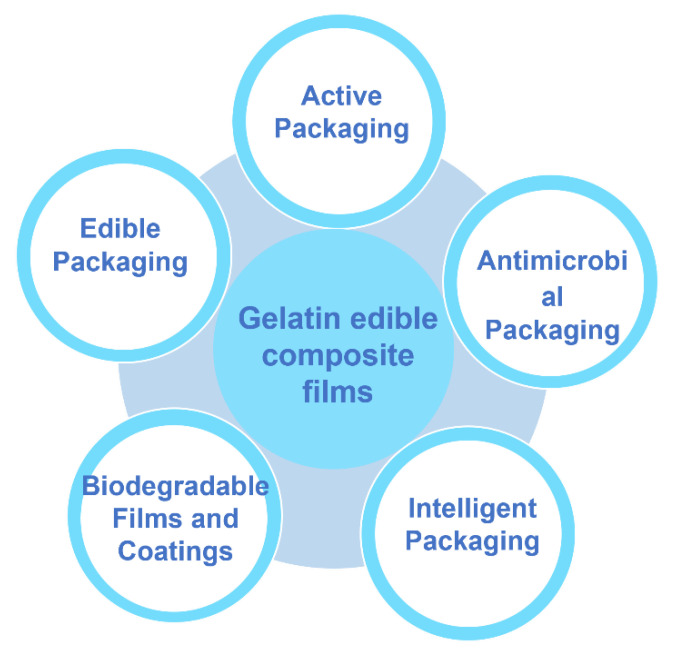
Various food packaging applications of gelatin.

In the process of picking, storage and transportation, the appearance and quality of fresh fruits are deteriorated due to the continuous production of ethylene ripening agents, such as over-ripening, water loss and decay. Edible films and coatings composed of gelatin-based materials have been evaluated to extend the shelf life of various fruits (Figure 9). A study reported that natural biological macromolecules gelatin, chitosan and CaCO_3_ integrated into multifunctional nano edible composite film for postharvest fruits (longan and banana) preservation. The results showed that the weight loss rate of fruits wrapped in edible multifunctional nanocomposite film was dramatically more reduced than the samples in the control group. The developed films exhibited excellent water resistance and antibacterial properties, and it could effectively extend the shelf life of longan for more than 3 days and bananas for more than 5 days, respectively [72]. Michelly et al. physically mixed cassava starch, gelatin, casein and sorbitol to prepare a thin film with low solubility and extremely low water vapor permeability. The degradation rate of chlorophyll in the coated fruit was delayed, leading to a less of a quality loss of the fruit and a high content of Vitamin C and soluble solid. Besides, the shelf-life of the guava fruit coated with this film was extended by two days. Coated fruit was still green after 9 days of storage, while uncoated fruit had lost its green color after 3 days of storage. It can be seen that gelatin-based blends delay fruit ripening and decay effectively [73].

### 5.2. The Vegetables

Gelatin is also used to preserve vegetables. Vegetables are rich in vitamins, minerals, fiber and other nutrients. These components play an important role in healthy dietary pattern. However, vegetables have a short shelf life due to their high moisture content (75–95%), leading to rapid spoilage and decay [74]. Fruits and vegetables regulate respiration rate to affect the concentration of O_2_ and CO_2_ to achieve relatively stable metabolic activities. Vegetables are sensitive to spoilage caused by mold, yeast and bacteria, so it is necessary to package and coat freshly picked vegetables [3]. Haiying et al. prepared chitosan nanoparticles containing clove oil (CO@CNPs) and gelatin composite membrane to preserve cucumber using the electrostatic spinning technique, and evaluated the number of *E. coli* on yellow flowers at different temperatures. The results showed that gelatin/CO@CNPs nanofiber membranes showed a higher efficacy on the inactivation of antibacterial than on pure gelatin films at low temperatures, compared to that in high temperature. After 4 days of storage, the appearance and the sensory scores of the foods in the experimental group were superior to those of the control group. Both color and taste were improved, compared with the control groups [75].

### 5.3. Aquatic Products

**Figure 10:**
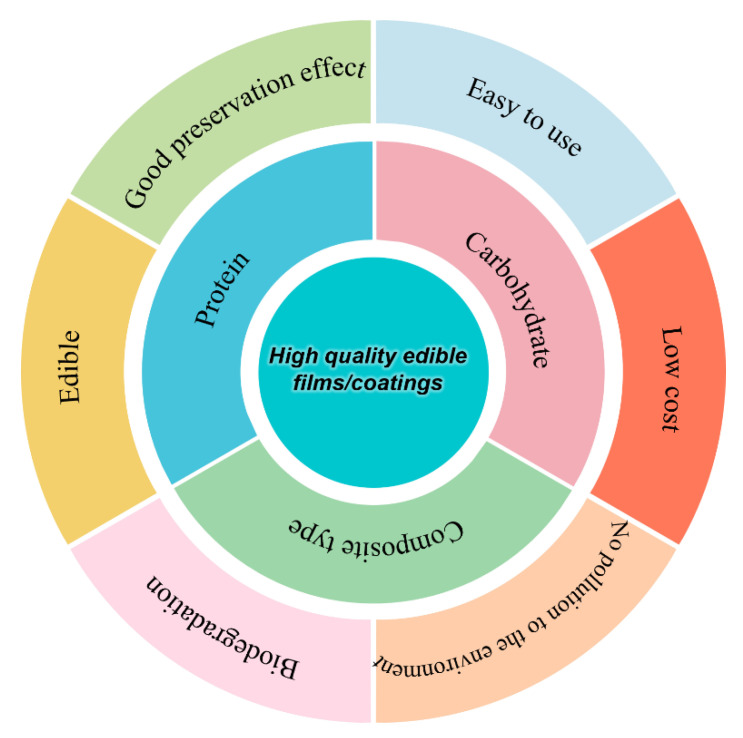
Characteristics of gelatin edible packaging films/coatings for high quality.

Aquatic products are perishable because they contain high contents of polyunsaturated fatty acids, proteins, free amino acids and endogenous enzymes, making them prone to oxidation and microbial degradation, resulting in discoloration, taste change and rancidity [76]. Edible coating is an effective way to improve product quality and extend the shelf life of aquatic products [77]. Gelatin is widely used in food packaging because of its good film forming and gelatinous properties, as well as its low aroma and good barrier properties [78] (Figure 10).

Xiong et al. developed a mixture coating of gelatin, chitosan, gallic acid and clove oil (GE-CH-GA-CO) to observe the fresh-keeping effect of fresh salmon fillet refrigerated at 4 °C for 15 days [79]. It was found that the mixture coating can effectively prevent the decrease of brightness of salmon fillet, which may be due to the antioxidant effect of the film itself on the surface of fish isolation and protection [78,80]. In addition, the gas, water vapor permeability of gelatin coating and the PH value of the all the coated fillet samples decreased, and the incorporation of GE–CH–CO enhanced the antioxidant and antibacterial effects significantly, leading to a shelf-life extension by at least 5 days [79]. Fang He et al. developed an edible unidirectionally permeable film (UPF) based on κ-Carrageenan and gelatin to store fresh grass carp fillets at 4 °C UPF had better mechanical strength and flatness, compared to single gelatin film. In addition, the WVP of UPF was reduced and water resistance was increased. The TVB-N of samples packed with UPF was lower than that in the control group during the first 6 days of storage, which suggests that UPF effectively inhibited microbial growth and extended the shelf life of grass carp fillets. On day 6, TVB-N values of the samples in the control group and the UPF group reached 16.56 mg/100 g and 15.87 mg/100 g, respectively, indicating a slight corruption of grass carp (acceptable medium corruption limit of grass carp was 15 mg/100 g [81]) [82].

### 5.4. Meat

Meat, a high-value nutritional product in the human diet, contains a variety of essential fatty acids required by the body. However, during the production, processing and storage, meat products are prone to spoilage and deterioration, and the action of microorganisms and endogenous enzymes will result in meat freshness changes [83]. Nowadays, gelatin-based edible films and coatings have been widely applied in the preservation of meat products to retard the growth of microorganisms and maintain the texture properties of meats by modifying gelatin-based packaging. I h, A et al. prepared a gelatin film incorporated with nanochitosan and essential oil to preserve chicken breast. The results showed that the preserved chicken breast meat exhibited desirable sensory properties such as color, taste and odor during the whole storage time, and the antimicrobial activity was enhanced significantly, compared with control and samples wrapped with pure poly lactic acid [84]. Bermudez-Oria et al. introduced hydroxytyrosol or 3,4-dihydroxyphenylglycol into a pectin-fish gelatin edible film for the preservation of raw beef meat, finding that the oxygen barrier and the antioxidant protection was stronger than samples packaged by pure fish-gelatin film, and lipid oxidation was reduced by 100% over 7 days [85].

### 5.5. Baking

Baked foods are abundant in nutrition (protein, fat, fiber, sugar) and species (bread, cakes, biscuits). They are easy to oxidize and breed mold, leading to product deterioration. Food spoilage caused by mold can lead to huge economic losses and produce toxic secondary metabolites (mycotoxins) that have an impact on human health. Wenjie, et al. developed a gelatin film incorporated with papain to maintain desirable characteristics of bread quality, embodied in fortifying the frozen dough bread’s quality and obtaining a larger bread volume. After 60 days of frozen storage, glutenin macropolymer depolymerization degree and thiol groups (-SH) content reduced significantly, without significant differences in the alteration of secondary structure and high-temperature stability, retarding the detrimental deterioration of frozen dough. In conclusion, gelatin with papain could protect frozen dough effectively [86,87].

## 6. Conclusions and Future Research Direction

Gelatin is an edible active material that can be used to prepare edible films and coatings. It is an effective material for preserving fresh food due to unique antibacterial and antioxidant properties. However, gelatin, with high water solubility and high viscosity, is greatly affected by natural weather conditions and air humidity, thus there are still great limitations in food packaging. For example, with the loss of soluble matter and water, the surface structure of gelatin film is destroyed and it is difficult to achieve the ideal fresh-keeping effect when preserving fruits, vegetables and aquatic products. To overcome the major limitations of pure gelatin film, which is easily soluble in water, researchers usually mix gelatin with the traditional biological polymer and incorporate it with some natural active substances to obtain modified edible composite films and coatings. The modified composite films have desirable physical and chemical properties (barrier property, pH, browning index, etc.) and biological properties (antimicrobial, oxidation resistance properties), contributing to solving some limitations of gelatin composite film and expanding the scope of application in food preservation and future commercialization. At present, gelatin film can be prepared by coating, casting, extruding and electrospinning, but the preparation of films by means of extrusion molding is still not a simple matter. For instance, the extrusion film, with strong viscosity, is easy to break and stretch out and not easy to collect material. So, we still need to undertake further exploration on squeeze film. In addition, due to socio-cultural, religious and health-related issues, mammals are rejected to a large extent. Aquatic gelatin (fish gelatin) is an important food-grade hydrocolloid isolated from aquatic sources and is considered an excellent alternative to mammalian gelatin. Of course, gelatin is compounded with protein, carbohydrate and phenolics, which is a promising biological material attracting great interest in recent decades, which can develop based-gelatin edible films and coatings with novel health-promoting properties, such as probiotics and prebiotics films. Thus, the biological materials can increase their market appeal as healthy food ingredients with desirable sensory properties, making them promising for fruits, vegetables, meat, fish and derivative products.

## Figures and Tables

**Table 1:** Properties of gelatin-based composite edible films.

Composite Materials	Physical Properties	Biological Properties	Reference
Carbohydrate			
Aloe vera gel	—Reduced water solubility and tensile strength —No significant effect on thickness, water vapor permeability (WVP) or color —The mechanical properties of aloe gel were decreased with the increase of aloe gel concentration —The plasticity was increased significantly	—Increased antioxidant properties	[30]
RNA	—Shielded ultraviolet ray (UV) radiation to the highest degree —Increased color change and browning index	—Increased antibacterial activity and antioxidant activity	[39]
Glucose	—Increased tensile strength, elongation at break and glass transition temperature —Increased UV resistance and water resistance (water solubility and water contact angle)	—Increased the antioxidant capacity significantly with the increase of glucose content —The antioxidant activity was increased to a lesser extent with increasing reaction time	[40]
Polyphenols			
Curcumin	—Increased the solubility of curcumin	—Increased antioxidant activity and inhibited the growth of pathogenic bacteria, *Streptococcus enterococcu Staphylococcus aureus*, *Listeria monocytogenes*, and *E. coli*	[41]
Protocatechuic acid (PCA)	—Increased thickness —Improved transparency and obtained fine appearance —Reduced light transmittance significantly —Reduced tensile strength and increased elongation at break	—High free radical scavenging activity of DPPH —Improved film stability and biological activity —Obtained good bacteriostatic effect with the increase of concentration of PCA	[42]
Polylactic acid, eugenol	—The internal fibers are uniform in shape —Enhanced surface water resistance and hydrophobicity —Improved encapsulation efficiency and load capacity	—Antioxidant and antibacterial activities were enhanced	[43]
Enzymes and proteins			
Microbial transglutaminase, gelatin-streptococcus, lactin/catechin	—Increased mechanical strength —Improved water vapor and UV resistance —Reduced the solubility and fluidity of films —Increased the viscosity of fish glue	—Increased the antibacterial and antioxidant activity to pathogenic bacteria —Prevented microbial growth and lipid oxidation of pork mince	[44]
Egg white protein	—Improved melting point and gel strength —Tensile strength, mechanical strength and deformation were significantly reduced —Improved UV barrier performance —Contact angle was significantly reduced, and surface wettability was increased	—Improved antibacterial and antioxidant properties	[45]
Other polymers			
Silver-kaolin	—Improved the surface morphology and structure —Increased the waterproof performance significantly —Increased the thickness and opacity —Reduced the flexibility and tensile strength —Lowed ultraviolet transmittance	—It showed significant inhibition against gram-negative bacteria and gram-positive bacteria (*E. coli*, *Staphylococcus aureus*, *Listeria monocytogenes* and *Salmonella typhimurium*)	[46]
Rutin functionalized cellulose nanocrystal (RCNC)	—Improved thermal stability, dispersion and compatibility —The highest UV-visible light and water vapor resistance —Improved tensile strength and antibacterial performance significantly with the addition of RCNC	—The antibacterial performance of *Staphylococcus aureus* and *E. coli* was improved	[47]
Cinnamon essential oil (CEO)	—The tensile strength, elongation at break and water content of gelatin-based films was decreased with the increase of CEO concentration, but water vapor permeability was increased. —Improved the light resistance —Improved the UV resistance	—Showed strong inhibition to various microbial pathogens —Enhanced the antibacterial and antioxidant properties of active films	[48]

**Table 2:** Findings and forming method of gelatin-based composite films and coatings on food preservation.

Foods	Composite Materials	Effects	Forming Method	Reference
Fresh fruit				
Strawberry	Probiotics, inulin	—Significantly reduced weight loss, water loss, respiration rate and delays decay —Slowed down pH change —Delayed titratable acidity (TA) content change —Significantly decreased the increase of total soluble solid (TSS) value —The total phenol (TPC) content and antioxidant activity were retained effectively —Inhibited the growth of yeast and mold	Coating	[62]
Fresh cut apple	Chitosan, tannin	—Reduced weight loss and malondialdehyde content —Delayed Browning —Inhibition of lipid oxidase activity —Improved oxidation resistance and barrier performance —Improved appearance quality	Mix dry	[63]
Banana	Lac	—Slowed chlorophyll degradation and aging, with slight color changes —Reduced titratable acidity, total soluble sugar content and weight loss —Significant increase in hardness —The total number of banana mold/yeast was increased slowly —Extended shelf life and maintained quality	Coating	[64]
Fresh vegetable				
Tomatoes	Titanium (Ti), nanoparticles (CuO)	—Greatly increased shelf life (up to 18 days at 40 ± 3 °C) —It had a higher antibacterial effect on gram-negative cells than gram-positive cells —The film was clean, transparent, shiny and flexible —Helped to maintain flavor, nutrition and color —Film biodegradability was enhanced	Casting	[65]
Aquatic products				
Abalone	Sodium alginate, plant extract (bamboo leaf extract, rosemary extract five)	—Kept good sensory characteristics —Inhibited microbial reproduction and endogenous enzyme activity —Extended the shelf life of abalone —Biogenic amine content was decreased —Decreased microbial population —Delayed pH to drop	Coating	[66]
Golden pompano piece	Chitosan	—Prevented myosin and myoglobin from degrading —Effectively controlled the weight loss, economic loss, retained the nutrition and original color —Significantly inhibited amino acid degradation and biogenic amine production —pH was more stable —Better anti-corrosion ability, antibacterial ability	Coating	[67]
Shrimp	Amaranth extract, quaternary ammonium chitosan	—Improved light blocking ability —Increased flexibility and oxidation resistance —Reduced stability and improved permeability —It had antibacterial and antioxidant capacity	Casting	[68]
Meat				
Meat emulsion	Grape seed oil, alginate	—Reduced the value of fat content, pH, firmness, chewiness, toughness, and lipid oxidation of the meat emulsion —Had a substantial effect on the physico-chemical properties of meat emulsion	Emulsion	[69]
Beef	Aqueous extracts of henna	—Preserved color properties significantly —Decreased the rate of proteolysis process —Decreased the lipid oxidation and microorganisms’ counts —Decrease weight loss and pH —Improve meat preservation	Coating	[70]
Baking				
Bread	Cashew gum, essential oil, ferulic acid	—Maintained bread quality characteristics —Delayed moisture loss and fungus growth —Storage period promoted six days	Casting	[71]

## Data Availability

Not applicable.
